# Efficacy of Cold Atmospheric Plasma Against Methicillin‐Resistant *Staphylococcus aureus* Biofilms: A Systematic Review of In Vitro Studies

**DOI:** 10.1155/bmri/4047432

**Published:** 2026-07-27

**Authors:** Reyhaneh Shoorgashti, Faezeh Dehghan Ghanatkaman, Sana Baghizadeh, Sarah Sadat Ehsani, Simin Lesan, Hooman Ebrahimi

**Affiliations:** ^1^ Researcher, Department of Oral Medicine, TeMS. C., Islamic Azad University, Tehran, Iran, azad.ac.ir; ^2^ Researcher, Faculty of Dentistry, TeMS. C., Islamic Azad University, Tehran, Iran, azad.ac.ir; ^3^ Department of Diagnosis and Oral Health, School of Dentistry, University of Louisville, Louisville, Kentucky, USA, louisville.edu; ^4^ Assistant Professor, Department of Oral Medicine, TeMS. C., Islamic Azad University, Tehran, Iran, azad.ac.ir

## Abstract

**Background:**

Methicillin‐resistant *Staphylococcus aureus* poses a serious threat to global health due to its resistance to conventional antibiotics and its ability to form resilient biofilms. Cold atmospheric plasma has emerged as a promising alternative for microbial biofilm inactivation.

**Objective:**

To systematically evaluate the in vitro efficacy of cold atmospheric plasma in disrupting or eradicating methicillin‐resistant *Staphylococcus aureus* biofilms and to identify factors influencing treatment outcomes

**Methods:**

A comprehensive search was conducted in five databases (PubMed/MEDLINE, Embase, Scopus, Web of Science, and Google Scholar) for in vitro studies published up to July 2025. Eligible studies assessed cold atmospheric plasma effects on methicillin‐resistant *Staphylococcus aureus* biofilms. Data were extracted on study characteristics, cold atmospheric plasma device parameters, exposure conditions, and microbial outcomes. Risk of bias was assessed using a modified version of the ToxRTool.

**Results:**

Seventeen in vitro studies were included. Most studies originated from European countries (*n* = 10), with a peak in publications observed in 2021. Dielectric barrier discharge and plasma jets were the most common devices. Air‐based plasmas were the most commonly used in 12 studies, followed by helium. All studies reported cold atmospheric plasma‐mediated reductions in methicillin‐resistant *Staphylococcus aureus* biofilm load, with log_10_ colony‐forming unit reductions ranging from 1 to > 6, depending on exposure time, surface material, and device configuration. Several studies demonstrated near‐complete biofilm eradication within minutes. Synergistic effects were observed when cold atmospheric plasma was combined with antibiotics.

**Conclusions:**

These findings suggest that cold atmospheric plasma may have potential as an adjunctive strategy for biofilm‐related infection control, although further standardized and clinically relevant studies are needed.

## 1. Introduction

*Staphylococcus aureus* (*S. aureus*) is a common bacterium that colonizes the skin and mucosal surfaces of humans [1–4]. Although often harmless, it can cause a wide range of infections when natural barriers are breached—for example, through surgical procedures or traumatic wounds [5, 6]. These infections can vary in severity, from minor skin conditions to serious invasive diseases such as bacteremia, endocarditis, osteomyelitis, and pneumonia [1, 7, 8]. Among the various strains of *S. aureus*, methicillin‐resistant *S. aureus* (MRSA) represents a significant clinical and epidemiological challenge due to its intrinsic resistance to *β*‐lactam antibiotics, resulting in elevated morbidity, prolonged hospitalization, increased healthcare costs, and higher mortality rates [3, 4, 9].

A key factor contributing to the persistence and treatment failure of MRSA infections is its ability to form biofilms [9–12]. Biofilms are complex microbial communities embedded in a protective extracellular matrix that adheres to both biological tissues and inert surfaces, such as catheters, implants, and wound beds [9, 10, 13, 14]. Within this structure, bacteria become significantly less susceptible to antibiotics, often requiring concentrations far above therapeutic levels to achieve the same effect as in free‐floating (planktonic) cells [9, 10, 13, 15, 16]. Factors such as impaired antibiotic diffusion, phenotypic heterogeneity, metabolic dormancy of persister cells, and the biofilm′s physical barrier collectively contribute to the failure of conventional therapies [11, 12, 17]. Moreover, biofilms protect bacteria from immune responses and promote the development of persister cells, which can survive antibiotic exposure and lead to recurrent infections [11, 12, 17]. This therapeutic impasse underscores the urgent need for innovative strategies capable of effectively targeting biofilm‐associated infections.

Among the most promising nonantibiotic alternatives is cold atmospheric plasma (CAP), a type of ionized gas that operates at room temperature and atmospheric pressure. It contains electrons, ions, neutral atoms, ultraviolet radiation photons, and a cocktail of reactive oxygen and nitrogen species (RONS) [18–20]. In contrast to hot plasmas, CAP′s thermal energy is low (typically ~30°C–50°C), so it can contact living tissue without burning it.

CAP is produced by applying high voltage to a gas at atmospheric pressure. Broadly, generators fall into direct and indirect discharge types. In a direct discharge (often a dielectric barrier discharge [DBD]), plasma ignites between two electrodes (one or both covered by dielectric) or between an electrode and the tissue itself. These DBD devices can use ambient air as the working gas and can treat broad, flat areas. In contrast, indirect devices create plasma in a flowing gas. For example, atmospheric pressure plasma jets (APPJ) use a noble carrier gas (typically helium, argon, or mixtures) between electrodes; the plasma then propagates out as a narrow jet that can target distant or curved surfaces. Helium or argon flows through a tube (often glass or quartz), sustaining a plasma plume at the nozzle. Other CAP sources include corona or glow discharges and experimental hybrid systems, but these are less common in practice. Hybrid devices combine micro‐discharges with a mesh electrode to create uniform plasma.

CAP generates a mix of short‐lived (e.g., atomic O, •OH, and ONOO^−^) and longer lived (hydrogen peroxide [H_2_O_2_] and NO_2_^−^/NO_3_^−^) reactive species. These RONS diffuse into cells and oxidize lipids, proteins, and nucleic acids. The oxidative stress causes membrane lipid peroxidation and deoxyribonucleic acid (DNA) damage, metabolic disruption, and loss of culturability [21–26].

The biological effects of CAP are dose‐dependent. Low doses tend to trigger cell signaling or arrest, whereas higher doses can induce cell death. Unlike traditional therapies, CAP has shown the ability to penetrate biofilms, disrupt the biofilm matrix, and reduce microbial viability [21–26].

Recent laboratory studies have demonstrated CAP′s potential in significantly reducing MRSA biofilms on various surfaces, suggesting possible applications in wound care, infection control, and device sterilization [2, 15, 16, 27–30]. However, despite growing interest in this technology, the evidence remains scattered and variable in quality. To date, there has been no comprehensive synthesis of available data specifically focused on the efficacy of CAP in disrupting MRSA biofilms.

This systematic review aims to address this gap by critically evaluating the existing literature on the use of CAP for the disruption or eradication of MRSA biofilms and to identify factors influencing treatment outcomes. By assessing the efficacy, limitations, and methodological quality of current evidence, this review seeks to inform both clinical practice and future research.

## 2. Methods

This systematic review was conducted in accordance with the Preferred Reporting Items for Systematic Reviews and Meta‐Analyses (PRISMA) 2020 guidelines [31]. Due to restrictions from the International Prospective Register of Systematic Reviews (PROSPERO) regarding the registration of in vitro studies, the protocol has not been registered.

### 2.1. Information Sources and Search Strategy

A systematic literature search was conducted in PubMed/MEDLINE, Embase, Scopus, and Web of Science to identify eligible studies published up to June 2025. In addition to these core databases, Google Scholar was also searched to ensure comprehensive coverage and to identify potentially missed studies. An updated search was conducted on July 27, 2025. The reference lists of included studies and relevant reviews were manually screened for additional records.

Database‐specific search strategies were constructed using a combination of Medical Subject Headings (MeSH) and free‐text terms related to “cold atmospheric plasma,” “CAP,” “biofilm,” and “MRSA.” Boolean operators and truncation were employed to capture all relevant variations (see Table S1).

### 2.2. Eligibility Criteria

This review aimed to answer the following research question using the PICO framework:

“In in vitro studies investigating biofilm‐forming MRSA, what is the efficacy of CAP treatment compared to no treatment or conventional antimicrobials in reducing viable MRSA biofilm load?”

• Population: In vitro studies evaluating biofilms formed by MRSA. Only studies using confirmed MRSA strains with biofilm formation were included.
• Intervention: Use of CAP in any form (e.g., plasma jets, DBD, or cold plasma‐activated solution [PAS]) for the purpose of MRSA biofilm disruption or inactivation
• Comparator: No treatment, conventional disinfection methods, or antibiotics
• Outcomes: Primary outcomes included quantitative changes in MRSA microbial load (e.g., log_10_ colony‐forming unit [CFU] reduction) before and after CAP exposure. Secondary outcomes included factors influencing treatment outcomes (e.g., gas composition and exposure time).

Only articles published in English were included. There were no restrictions on the year of publication or the country of origin.

Exclusion criteria include in vivo studies, reviews, editorials, conference abstracts, and studies that do not report quantifiable outcomes related to MRSA biofilm inactivation.

### 2.3. Study Selection

All search results were imported into EndNote X9 (Clarivate) for reference management. Duplicate entries were removed. Two reviewers (S.B. and S.E.) independently screened titles and abstracts for potential relevance. Full‐text versions of shortlisted studies were then reviewed against the eligibility criteria. Any disagreements were resolved through discussion or with input from a third reviewer (R.S.).

### 2.4. Data Extraction

Two reviewers (F.D. and S.B.) independently extracted relevant data from each eligible study using a standardized form. Extracted information included bibliographic details (authors, publication year, and country of origin) and study objectives. Bacterial characteristics were recorded, with specific attention to the *S. aureus* strain used and methods employed to confirm MRSA biofilm formation. For the intervention, detailed technical specifications of the CAP device were documented, including device type, gas composition, applied voltage, frequency, exposure duration, and treatment intensity. Comparator groups were also characterized, noting whether they involved antimicrobial agents, alternative disinfection strategies, or untreated controls. Primary outcome measures included quantitative indicators of biofilm reduction such as CFU counts, log_10_ reductions, and biomass quantification. Secondary outcomes encompassed viability assays, eradication rates, and any adverse effects associated with CAP exposure.

Any disagreements were resolved through discussions with a third reviewer (R.S.).

### 2.5. Risk of Bias Assessment

The risk of bias in individual studies was evaluated using a modified version of ToxRTool [32, 33], adapted for in vitro biofilm studies evaluating CAP. The assessment framework comprised five domains: (1) test substance characterization, (2) test system characterization, (3) study design, (4) results documentation, and (5) data plausibility.

The test substance characterization domain evaluated the adequacy of CAP device reporting, including device type, operating parameters, gas composition, and treatment conditions. The test system characterization domain assessed the description of the biofilm model, including bacterial strain identification, substrate characteristics, biofilm growth conditions, and biofilm maturity. The study design domain evaluated the use of appropriate controls, replication procedures, and methodological transparency. Results documentation assessed the completeness and clarity of outcome reporting and statistical analyses. Data plausibility assessed the consistency and credibility of the reported findings with respect to the described methodology. Detailed domain‐level assessments are provided in Table S2.

Risk of bias assessments were performed independently by two reviewers (R.S. and S.L.), with disagreements resolved through discussion and consultation with a third reviewer (H.E.) when necessary.

## 3. Results

### 3.1. Study Selection

**Figure 1:**
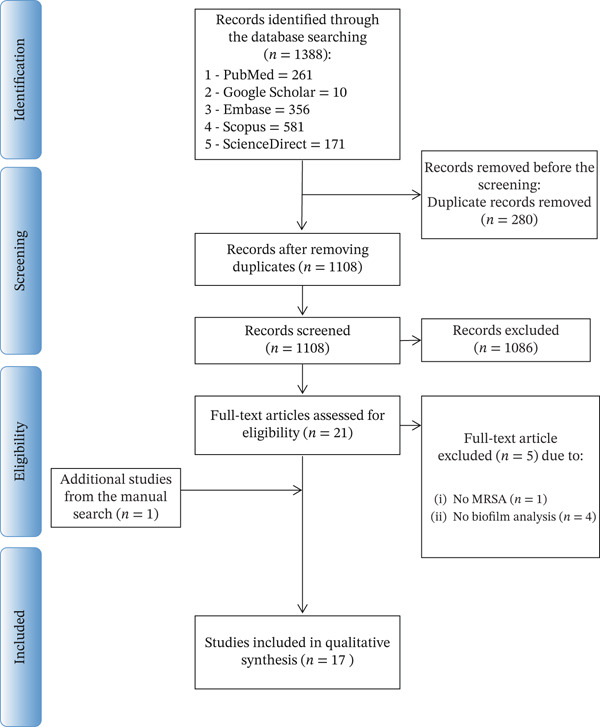
PRISMA flow diagram of study selection. Flowchart depicting the identification, screening, eligibility, and inclusion of studies based on PRISMA 2020 guidelines.

A total of 1388 records were identified through systematic searches of five electronic databases. After removing 280 duplicates, 1108 records underwent title and abstract screening. Of these, 21 full‐text articles were assessed for eligibility, with 17 studies meeting the inclusion criteria for qualitative synthesis. The study selection process is summarized in the PRISMA flow diagram (Figure 1).

### 3.2. Study Characteristics

**Figure 2:**
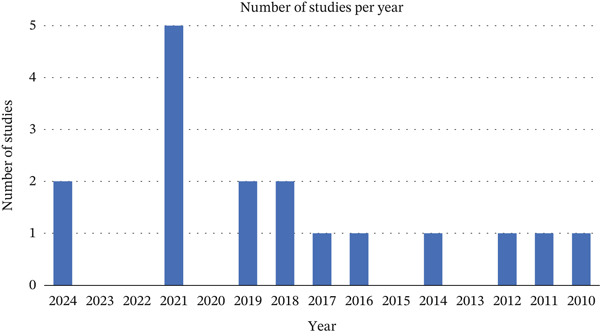
Annual distribution of included studies. Number of in vitro studies on CAP for MRSA biofilms published annually from 2010 to 2024, with the highest publication rate in 2021 (*n* = 5).

**Figure 3:**
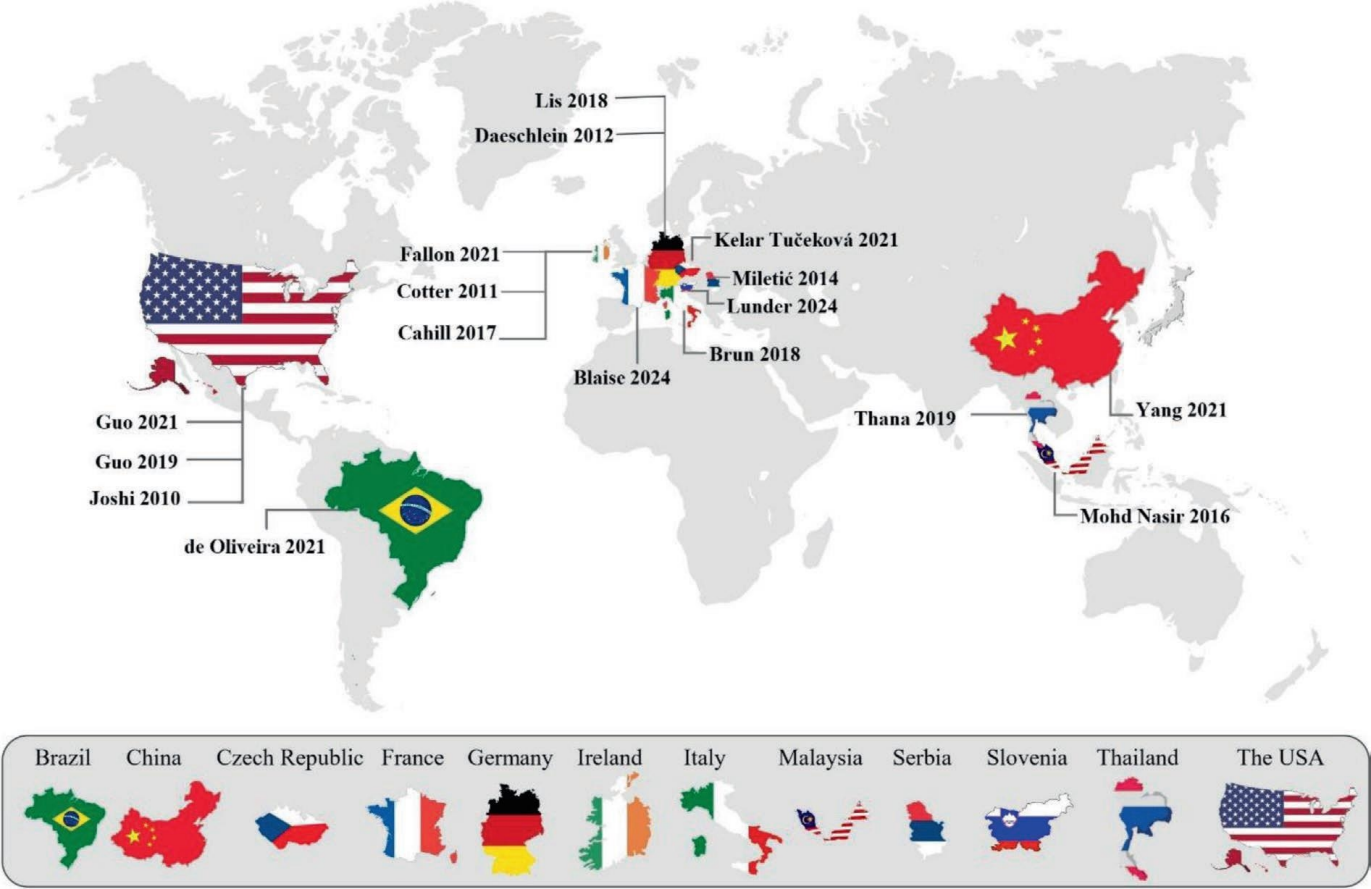
Geographic distribution of studies. Countries of origin of included studies. The United States and Ireland contributed the most (three studies each), with European countries contributing the majority overall (*n* = 10).

Seventeen in vitro studies published between 2010 and 2024 were included (Figure 2). Publication trends showed an increase over time, peaking in 2021 (*n* = 5) [16, 28–30, 34], whereas no eligible studies were identified in 2013, 2015, 2020, or 2022. Geographically, studies originated from 12 countries, with the United States [29, 35, 36] and Ireland [16, 37, 38] contributing the most (*n* = 3 each), followed by Germany (*n* = 2) [39, 40]. Remaining studies came from Brazil [28], China [30], France [15], Czech Republic [34], Italy [41], Malaysia [42], Serbia [43], Slovenia [2], and Thailand [44] (Figure 3) (see Tables S2 and S3).

### 3.3. MRSA Strain Distribution and Biofilm Maturity Confirmation

Across the included studies, a wide range of MRSA strains was investigated, demonstrating substantial strain heterogeneity. Five studies evaluated the reference strain ATCC33591, representing the most frequently used isolate [28–30, 35, 44]. Three studies each investigated ATCC 43300 [2, 34, 38] and the community‐associated strain USA300 [15, 36, 39]. Two studies used the BH1CC strain [16, 37]. Three studies did not report the MRSA strain [40, 42, 43]. Single studies investigated USA400, ATCC 33592 [36], hospital‐associated MRSA (HA‐MRSA) [39], and livestock‐associated MRSA (LA‐MRSA) [39].

For confirmation of biofilm maturity, most studies did not directly assess biofilm maturation and relied primarily on indirect indicators such as incubation time and CFU measurements (*n* = 11). Where maturation was evaluated, confocal scanning laser microscopy was the most frequently used technique (*n* = 3) [15, 16, 39], followed by fluorescence microscopy (*n* = 2) [2, 40]. A single study used multiphoton microscopy [15], and one study employed scanning electron microscopy (SEM) [28].

### 3.4. CAP‐Generating Devices and Treatment Parameters

**Figure 4:**
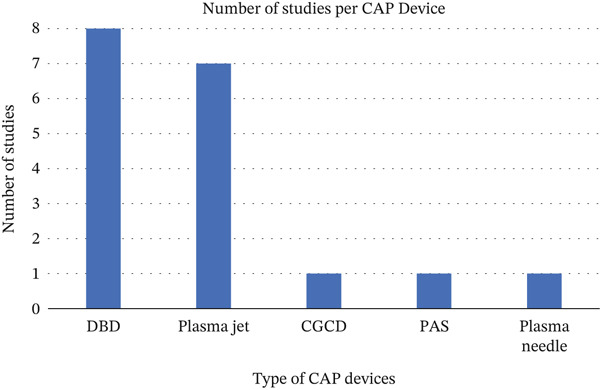
Distribution of cold atmospheric plasma device types used in the included studies.

A diverse range of CAP‐generating devices was reported (Figure 4). DBD systems were most frequently employed (*n* = 8) [29, 34–37, 39, 40, 42], followed by plasma jets (*n* = 7) [2, 15, 16, 28, 38, 41, 44], and single studies utilizing corona‐generated cold discharge (CGCD) [42], plasma‐activated saline (PAS) [30], and plasma needle technologies [43]. Exposure durations varied substantially, ranging from 15 s to 90 min, although most studies implemented short treatment times (≤ 5 min), reflecting an emphasis on clinically translatable disinfection windows.

### 3.5. Plasma Gas Compositions

Working gas compositions varied considerably across the included studies. Fourteen studies employed a single gas composition for CAP generation [2, 15, 16, 28, 30, 36–44]. Among these, air‐based plasmas were the most frequently used (*n* = 9) [2, 16, 36–40, 42, 44]. Helium was used as the sole working gas in four studies [15, 28, 41, 43]. Also, one study did not report the gas composition [30].

Three studies evaluated multiple gas compositions within the same experimental design [29, 34, 35]. Two studies by Guo et al. compared several gas mixtures, including argon supplemented with synthetic air, helium supplemented with synthetic air, natural air, and synthetic air alone [29, 35]. Another study by Kelar Tučeková et al. compared ambient air and water vapor as feed gases [34]. The diversity of gas compositions and gas mixture strategies reflects substantial heterogeneity in CAP generation approaches, which may contribute to variations in reactive species production and antimicrobial activity against MRSA biofilms.

The variability in gas types underscores the heterogeneity in CAP generation and its possible influence on antimicrobial efficacy.

### 3.6. Efficacy of CAP Against MRSA Biofilms

All included studies reported antimicrobial efficacy of CAP against MRSA biofilms, although reductions varied based on device type, exposure duration, biofilm maturity, and substrate material. Results are synthesized thematically based on the experimental model.

#### 3.6.1. Tissue‐Equivalent Models

Two studies utilized tissue‐representative substrates [15, 28]. Blaise et al. applied helium‐based DBD plasma to three‐dimensional (3D) bioengineered human wound models, achieving a 0.83 log_10_‐CFU/mg reduction after 2 min [15]. Similarly, de Oliveira et al. demonstrated a 0.93 log_10_‐CFU/mL reduction on collagen membranes after 5 min [28]. Both studies highlight CAP′s potential in wound‐related applications, although efficacy plateaued despite longer exposure, suggesting possible resistance thresholds in complex tissue‐mimetic systems.

#### 3.6.2. Culture‐Based Biofilm Models

Three studies evaluated CAP efficacy on biofilms grown on agar or standard culture media [2, 42, 44]. Lunder et al. demonstrated consistent 1 log10‐CFU/mL reductions in biofilms of different ages (24–72 h), suggesting maturity‐independent effects [2]. Thana et al. reported total eradication (> 6 log_10_) within 120 s using an ambient air plasma jet [44]. In contrast, Mohd Nasir et al. found marked differences between systems: CGCD achieved 9 log10‐CFU/mL reductions within 30 min, whereas DBD led to only 1 log_10_ reduction under identical conditions [42]. These findings emphasize the importance of device configuration and plasma generation dynamics.

#### 3.6.3. Microplate‐Based Biofilm Assays

Seven studies employed microplate‐based models to quantify CAP efficacy against MRSA biofilms under controlled in vitro conditions [16, 28–30, 35, 36, 41, 43]. De Oliveira et al. demonstrated that CAP exposure reduced MRSA biofilm burden from 8.81 to 5.90 log_10_ CFU/mL within 5 min, although mild regrowth was observed by the 7‐min mark, suggesting a time‐sensitive window of maximum efficacy [28]. Guo et al. reported a 3.5 log10‐CFU/mL reductions in bacterial load after 6 min of treatment with a CAPP system, highlighting the relevance of exposure duration in determining efficacy [29].

In another study, Yang et al. explored the use of PAS alone and in combination with antibiotics [30]. Although PAS monotherapy reduced bacterial counts to approximately 7.1 log_10_ CFU/mL, combining PAS with vancomycin or rifampicin achieved reductions below 2 log_10_, indicating substantial synergistic effects and potential for combinatorial strategies. Guo et al. used surface discharge plasma generated with either helium or argon and observed reductions of up to 5 log_10_ CFU/mL within 5 min, demonstrating that gas composition significantly influenced antimicrobial outcomes [35].

Brun et al. applied a helium‐driven radiofrequency plasma jet and achieved 1.5‐2 log10‐CFU/mL reductions after just 2 min of exposure [41]. Similarly, Miletić et al. and Joshi et al. reported near‐complete eradication of MRSA biofilms within 120 s using plasma needle and floating‐electrode DBD systems, respectively [36, 43]. These findings reinforce that CAP can produce rapid and dose‐dependent antimicrobial effects in microplate models.

#### 3.6.4. Hospital‐Relevant Surface Models

Seven studies assessed the efficacy of CAP against MRSA biofilms on materials that simulate clinical and hospital environments [16, 34, 36–40]. On glass surfaces, Fallon et al. observed a decrease in bacterial viability from 43%–56% in untreated controls to just 7%–18% following 1.5 min of CAP treatment, whereas Cotter et al. demonstrated >  5 log10‐CFU/cm^2^ reductions after 90 min of exposure [16, 37].

On polymeric materials, such as polypropylene textiles and polyethylene, CAP exposure resulted in reductions ranging from 2.9 to 5 log_10_, as reported by Kelar Tučeková et al. and Daeschlein et al., respectively [34, 39]. Stainless steel, commonly used in surgical tools and medical surfaces, was also effectively decontaminated. Cahill et al. achieved over 6 log10‐CFU/mL reductions within just 45 s, whereas Lis et al. showed a clear time‐dependent effect, reaching a maximum reduction of 3.38 ± 0.62 log_10_CFU/cm^2^ after 20 min [38, 40]. Other studies, including those by Fallon et al. and Daeschlein et al., corroborated the efficacy of CAP across various steel types [16, 39].

In addition, linoleum, a typical hospital flooring material, showed a notable reduction in viable MRSA cells to approximately 30% within 1.5 min of CAP exposure [16]. On hospital fabrics, such as mattress covers, CAP was similarly effective, with Cahill et al. reporting a 4.14 log10‐CFU/mL reduction in under 1 min [38].

### 3.7. Exposure‐Time Dependent Reduction Patterns

Many studies showed increased MRSA inactivation with longer CAP exposure [15, 29, 35, 37, 40]. However, Lunder et al. reported a nonlinear trend. Using a gliding arc plasma jet, the highest reductions in 24‐, 48‐, and 72‐h biofilms were observed after 1 min of treatment (3.54, 5.37, and 4.91 log_10_ CFU/cm, respectively), with decreasing efficacy at 2 and 3 min [2]. A similar biphasic pattern was noted by de Oliveira et al., where MRSA burden in collagen‐based biofilms declined from 8.81 to 5.90 log_10_ CFU/mL over 5 min, but slightly increased to 6.15 log_10_ CFU/mL at 7 min [28]. These findings suggest that extended exposure does not always correspond to increased efficacy in certain biofilm contexts.

### 3.8. Risk of Bias Assessment

**Figure 5:**
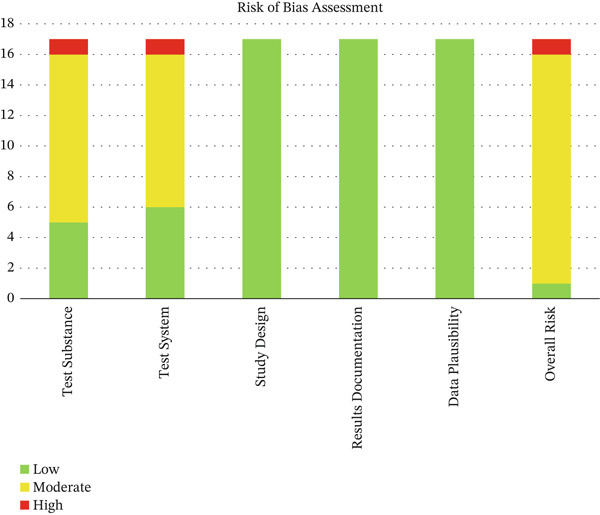
Risk of bias assessment across studies.

Risk of bias was assessed using a modified version of the ToxRTool, covering five domains (Figure 5). All 17 included studies were rated as low risk for study design, result documentation, and data plausibility. Greater variability was observed in the test substance and test system domains. For the test substance domain, five studies were rated as low risk, 11 as moderate risk, and one as high risk. Six studies were rated as low risk, 10 as moderate risk, and one as high risk for the test system domain. These ratings primarily reflected insufficient characterization and justification of the in vitro biofilm models, as well as limited technical reporting of CAP device specifications and operating parameters. The complete item‐level risk‐of‐bias assessments for each included study are provided in Table S5.

## 4. Discussion

This systematic review evaluated the in vitro efficacy of CAP against MRSA biofilms and analyzed the factors influencing treatment outcomes. Despite the considerable heterogeneity in experimental designs, plasma device types, exposure times, and gas compositions, all included studies demonstrated that CAP exerts measurable and often substantial antimicrobial effects against biofilm‐embedded MRSA.

CAP demonstrated substantial antimicrobial activity against MRSA biofilms across a range of in vitro experimental models. Several included studies reported complete or near‐complete biofilm eradication following relatively short treatment durations, achievements rarely matched by antibiotic monotherapy [36, 39, 42–46]. Traditional antibiotics often exhibit limited penetration and efficacy within established biofilms due to diffusion barriers and metabolic dormancy of sessile cells [46–51]. However, CAP physically and chemically disrupts the extracellular matrix and induces oxidative stress through the generation of RONS [19, 21, 22].

CAP‐generated RONS can traverse wound environments (liquid exudate, necrotic debris) and interact with microbes and host biomolecules [52–55]. In vitro and ex vivo models showed that RONS (especially long‐lived species like H_2_O_2_ and nitrites) can diffuse through aqueous layers or gels, enabling microbial kill beneath surface layers [52]. In real wounds, evidence is limited, but in animal and clinical wound models, CAP applied to burn eschar or slough did not impair healing and even reduced eschar thickness [19, 54, 55]. CAP has been observed to coagulate exudate proteins into a thin nanofilm (50–70 nm) that can act as a barrier against microbes [55].

In chronic wound exudate, CAP treatment upregulated growth factors (FGF‐2 and VEGF‐A) and pro‐inflammatory cytokines (TNF*α*, IL‐1*α*, and IL‐8) without raising total protein or matrix metalloproteinase levels, indicating selective modulation rather than wholesale protein denaturation [15, 56]. So, CAP might be able to penetrate wound exudates and necrotic layers to deliver antimicrobial effects without significant host tissue damage [15, 19, 55, 56]. However, quantification of exact penetration depths and detailed protein alterations in situ remain sparse. So, future studies should use sensitive assays (e.g., mass spectrometry of oxidized amino acids, depth‐resolved imaging) on ex vivo human wound tissue to fill these gaps.

Compared with established antimicrobial strategies such as chlorhexidine, UV‐C irradiation, and sodium hypochlorite, CAP has been evaluated as an emerging physical modality for biofilm control; however, direct head‐to‐head comparative evidence under standardized conditions remains limited, and available findings should therefore be interpreted within the constraints of substantial methodological heterogeneity. For instance, CAP has shown antibacterial activity similar to 0.2% chlorhexidine against *Enterococcus faecalis* biofilms and may be more effective in reducing the pathogenicity of mature oral biofilms when compared with lower chlorhexidine concentrations (0.12%) while avoiding well‐known side effects such as tooth discoloration and mucosal irritation [57, 58].

Experimental comparisons with UV‐C systems indicate that DBD plasma can achieve similar energy efficiency per treated area, with the additional advantage of reduced shadowing effects, allowing treatment of irregular or complex surfaces. Plasma‐based systems, however, may show reduced efficacy on dry surfaces, suggesting that moisture can influence reactive species delivery [59].

In comparison to sodium hypochlorite, sodium hypochlorite remains one of the most effective agents for rapid biofilm decontamination. However, its clinical use is limited by cytotoxicity, tissue irritation, and harmful by‐product formation. CAP can achieve comparable antibacterial effects without leaving chemical residues and may act synergistically with sodium hypochlorite by enhancing antimicrobial activity and enabling the use of lower chemical concentrations. In dentistry, short CAP exposure has produced reductions of up to 99.999% in *E. faecalis* biofilms, with decreases in bacterial viability and metabolic activity comparable to 6% sodium hypochlorite. Although sodium hypochlorite remains more effective for rapid bacterial elimination, CAP plasma jets can achieve substantial bacterial reduction after approximately 5 min of treatment, approaching the efficacy of conventional irrigants [60, 61].

Synergistic interactions between CAP and antibiotics were also consistently reported [30, 62–66]. When combined with vancomycin or rifampicin, PASs achieved eradication levels exceeding 5 log_10_‐CFU/mL reductions, far surpassing either intervention alone [30]. This synergy likely arises from plasma‐induced disruption of biofilm architecture, enhanced antibiotic penetration, and stress sensitization of bacterial membranes [62, 64, 66, 67].

The antimicrobial efficacy of CAP is influenced by several factors, including the type of plasma device used, the gas composition utilized for plasma production, the surface biofilm that was cultured and exposed to CAP, and the duration of CAP exposure.

Device geometry and energy delivery mechanisms can play critical roles in accelerating microbial kill kinetics. Systems such as the CGCD required extended exposures (up to 30 min) to achieve complete MRSA eradication [42]. However, other configurations, like plasma needle systems and floating‐electrode DBDs, achieved near‐complete or complete MRSA biofilm clearance within 2 min [36, 43].

The type of gas utilized in the production of CAP is another important factor. Noble gases such as helium and argon are widely favored in plasma physics for their stability and ability to sustain low‐temperature discharges enriched with RONS [68–70]. However, the findings of this review do not consistently support their superiority over air‐based CAP systems in antimicrobial performance. Several air‐based systems, particularly those using ambient or synthetic air in plasma jets or DBD configurations, achieved equivalent or greater MRSA biofilm inactivation in shorter times [29, 34, 36, 39, 42, 44].

For instance, Thana et al. reported complete eradication of MRSA biofilms on bacterial cultures within 2 min using an ambient air CAP jet [44]. Similarly, Joshi et al. achieved complete inactivation on 96‐well plates within 2 min using a floating‐electrode DBD system operating in air [36]. Daeschlein et al. also demonstrated full bacterial clearance, including MRSA, on stainless steel using an air‐fed DBD device with only 1 min of exposure [39].

In contrast, helium‐based CAP configurations, particularly those applied to more biologically complex surfaces, often achieved more modest reductions within comparable exposure times. For example, Blaise et al. and de Oliveira et al., both using helium‐fed DBD systems on collagen membranes and 3D skin models, reported only 1–2 log10‐CFU/mg reductions after 1–2 min of treatment [15, 28]. Similarly, Brun et al. achieved a 1.5‐2 log10‐CFU/mL reduction in 2 min on 96‐well plates using a helium radiofrequency plasma jet, and Guo et al. reported up to 5 log10‐CFU/mL reduction in 5 min on 24‐well plates using helium‐ or argon‐based surface discharge CAP [35, 41]. These data can suggest that, when used on nonporous surfaces, air‐based CAP systems can be highly effective and may rival or outperform noble gas‐based systems.

In addition to the CAP device and the utilized gas, the nature of the substrate on which MRSA biofilms were cultured can play a substantial role in determining CAP efficacy [71–73]. The included studies examined a wide range of materials, from inert, nonporous surfaces such as stainless steel [16, 38–40], glass [16, 36, 37], and polyethylene [34, 39], to biologically relevant substrates like collagen membranes [28] and engineered 3D skin models [15]. CAP treatments generally showed higher efficacy on smooth, nonabsorbent surfaces. For example, Daeschlein et al. and Cahill et al. demonstrated complete or near‐complete MRSA inactivation on stainless steel within 1 min, likely due to reduced diffusion barriers and optimal plasma–biofilm contact [38, 39].

In contrast, biofilms formed on tissue‐mimetic surfaces demonstrated more variable and often attenuated responses to CAP. In studies using collagen‐rich or hydrated wound models [15, 28], log reductions were limited to approximately 1 log_10_ even after prolonged exposure. This diminished response may be due to surface topography and the presence of extracellular organic matter that can scavenge reactive species or hinder plasma penetration [49, 74–77]. These findings underscore the importance of considering substrate complexity when evaluating CAP efficacy and highlight the need to tailor treatment parameters to the clinical context, whether disinfecting surgical instruments or treating biofilm‐infected wounds.

Even environmental factors (e.g., humidity, ambient airflow, and oxygen concentration) can substantially modulate CAP chemistry and thus affect antimicrobial results [78–81]. Airflow (ventilation or shielding) can alter gas‐phase species: A stagnant environment retains RONS, whereas airflow or shielding gas can remove or concentrate them [79, 82]. Spectroscopic evidence demonstrates that even small increases in feed gas humidity (below 1000 ppm) can markedly enhance hydroxyl (OH) radical emission, indicating a strong sensitivity of plasma chemistry to moisture content. Moreover, increased feed gas humidity has been associated with a near‐linear rise in H_2_O_2_ concentrations in treated liquids, which correlates with reduced cell viability through oxidative stress mechanisms [79].

Importantly, the relative contribution of ambient humidity depends on feed gas conditions: When humidified feed gas is used, ambient humidity has minimal influence, whereas under dry gas conditions, ambient humidity becomes a critical determinant of reactive species generation. In addition, technical factors such as gas delivery systems may introduce uncontrolled variability; for example, polymeric tubing can allow substantial diffusion of water vapor, resulting in significantly higher humidity levels compared to metal tubing and leading to unstable plasma conditions over time [79].

Exposure duration was consistently identified as a critical determinant of CAP efficacy in the in vitro studies reviewed. Although most studies in this review demonstrated a positive correlation between CAP exposure time and MRSA biofilm reduction, one notable exception was reported by Lunder et al. [2] In this study, longer exposure times resulted in progressively lower log_10_ reductions across biofilms of varying maturities. For example, in 24‐h MRSA biofilms, a 3.54 log10‐CFU/cm^2^ reduction was achieved after 1 min, compared to just 1.46 and 0.59 log10‐CFU/cm^2^reductions after 2 and 3 min, respectively. A similar decline in efficacy with time was observed in 48‐ and 72‐h biofilms. Similarly, de Oliveira et al. found that MRSA counts decreased from 8.81 to 5.90 log_10_ CFU/mL over 5 min, followed by a slight increase to 6.15 log_10_ at 7 min [28].

Several complementary mechanisms may explain the paradoxical reduction in antimicrobial efficacy observed with prolonged CAP exposure. First, plasma chemistry is inherently time‐dependent. During extended treatment, short‐lived RONS can undergo recombination, quenching, or conversion into less reactive species, thereby reducing the effective oxidative flux delivered to the biofilm [83, 84]. In addition, prolonged plasma treatment can modify local environmental conditions (e.g., humidity, temperature, pH, and electrical conductivity), which may alter plasma–surface coupling and decrease the generation or penetration of reactive species over time [67, 85, 86].

Second, prolonged sublethal oxidative stress may induce adaptive biofilm stress responses. Bacterial biofilms are known to respond to oxidative and nitrosative stress by upregulating antioxidant enzymes (e.g., catalase and superoxide dismutase), efflux pumps, DNA repair systems, and extracellular polymeric substance (EPS) production. Increased EPS synthesis can enhance biofilm density and act as a scavenging barrier that neutralizes reactive species before they reach deeper biofilm layers. This protective response may reduce susceptibility during extended exposure [83, 84, 87, 88].

Third, CAP exposure may preferentially eliminate metabolically active cells during early treatment while leaving behind persister cells and dormant subpopulations that are intrinsically more tolerant to oxidative stress. These surviving cells may subsequently resuscitate or regrow during longer treatment windows, producing the appearance of reduced efficacy or slight increases in CFU counts, as observed by Lunder et al or de Oliveira et al. [2, 28]

Finally, methodological factors may also contribute. CFU assays quantify only culturable cells and may underestimate sublethal injury or viable‐but‐nonculturable states. Transient biofilm dispersal followed by reattachment during longer exposure periods may further contribute to nonlinear killing curves.

Strain variability is a critical determinant of the observed heterogeneity in antimicrobial susceptibility and virulence among MRSA isolates, and this must be considered when interpreting CAP efficacy [89–91]. Community‐associated lineages such as USA300 and USA400 are widely recognized for enhanced virulence, largely attributed to the frequent carriage of Panton–Valentine leukocidin (PVL), elevated toxin production, and increased biofilm‐forming capacity, which can contribute to greater persistence and tolerance to antimicrobial stress [91, 92]. In contrast, classical healthcare‐associated strains such as ATCC 43300, ATCC 33591, and ATCC 33592 are typically characterized by broader multidrug resistance profiles and thicker, more structured biofilms, which may confer increased protection against disinfectants and physical decontamination approaches. Livestock‐ and HA‐MRSA lineages also demonstrate distinct phenotypic adaptations, including altered adhesion, metabolic flexibility, and stress response pathways, all of which may influence susceptibility to plasma‐generated reactive species [91–93]. Consequently, differences in virulence factors, biofilm architecture, and resistance mechanisms across strains can also contribute to the variability in reported CAP outcomes.

The results of this review suggest that CAP may represent a promising nonthermal antimicrobial approach for the reduction of MRSA biofilms under controlled laboratory conditions, particularly on medical devices, surgical instruments, and hard‐to‐clean surfaces in healthcare environments. However, because all included studies were conducted in vitro, the observed antimicrobial effects cannot be directly extrapolated to clinical settings. Additional investigations using tissue‐mimetic models, animal studies, and well‐designed clinical studies are required to determine the effectiveness, safety, and practical applicability of CAP for managing biofilm‐associated infections in patients.

### 4.1. Study Limitations

Despite the encouraging results, the review identified several limitations that restrict broader generalization. The review was restricted to English‐language publications, introducing the potential for language bias. Furthermore, due to high interstudy variability in outcome reporting (e.g., CFU vs. percentage reduction and different exposure times), statistical pooling of data was not feasible. The included studies employed a wide range of plasma sources, each characterized by distinct discharge physics and reactive species profiles. Direct comparison between studies is further complicated by inconsistent reporting of key treatment parameters such as power density, treatment distance, gas flow rate, temperature, and RONS flux. In many cases, studies reported applied voltage or exposure duration without providing quantitative measurements of delivered energy or reactive species concentrations. As a result, normalization of treatment intensity across studies was not possible, limiting the ability to directly compare antimicrobial efficacy between different CAP systems.

Another limitation of this study arises from the nature of in vitro analysis. Although focusing on in vitro studies provides valuable mechanistic insights, the findings may not fully translate to in vivo systems, where tissue interactions, immune responses, and environmental factors introduce additional complexity.

Microplate‐based biofilm models provide standardized and highly reproducible platforms for evaluating antimicrobial efficacy under controlled laboratory conditions. However, these systems represent simplified in vitro environments and do not replicate the structural and biological complexity of clinical wounds. Real wound biofilms develop within a heterogeneous microenvironment that includes extracellular matrix components, host immune factors, tissue architecture, oxygen and nutrient gradients, and wound exudate. These factors can influence biofilm structure, metabolic activity, and susceptibility to oxidative stress. So, antimicrobial effects observed on plastic microplates may overestimate clinical performance. Translation of CAP into wound care, therefore, requires validation in tissue‐mimetic models, animal studies, and clinical investigations. Also, most studies assessed short‐term bactericidal effects without evaluating long‐term regrowth potential or the effects of repeated CAP exposures.

An additional limitation concerns biofilm characterization and assessment of biofilm maturity. Although all included studies employed established incubation protocols to promote biofilm formation, only a minority confirmed biofilm architecture using microscopic techniques such as SEM or confocal laser scanning microscopy (CLSM). Furthermore, quantitative assessment of EPS, biofilm biomass, or structural organization was infrequently performed. Consequently, in many studies, the presence and maturity of biofilms were inferred primarily from incubation duration and CFU measurements, which may not reliably distinguish mature structured biofilms from dense bacterial aggregates or surface‐associated bacterial populations. Because susceptibility to CAP may vary according to biofilm architecture, thickness, and EPS composition, the reported antimicrobial effects should be interpreted as reductions in biofilm‐associated bacterial burden rather than definitive evidence of complete mature biofilm eradication unless structural confirmation was provided in the original studies.

## 5. Conclusion

Across diverse in vitro experimental models, CAP demonstrated antimicrobial activity against MRSA biofilms, although the magnitude of biofilm reduction varied considerably according to device configuration, treatment parameters, gas composition, substrate characteristics, biofilm maturity, and bacterial strain. Several studies reported complete biofilm eradication under specific experimental conditions; however, substantial methodological heterogeneity limits direct comparison across studies and prevents identification of optimal treatment protocols.

The available evidence supports the potential of CAP as an antimicrobial approach for reducing MRSA biofilms in vitro, particularly when combined with antimicrobial agents. Nevertheless, the findings of this review are limited to laboratory‐based studies and should not be interpreted as evidence of clinical effectiveness. Further research using standardized experimental protocols, clinically relevant biofilm models, animal studies, and human investigations is required before conclusions regarding clinical efficacy can be drawn.

## Author Contributions

**Reyhaneh Shoorgashti**: conceptualization, data curation, formal analysis, investigation, methodology, visualization, project administration, software, validation, writing—original draft, writing—review and editing. **Faezeh Dehghan Ghanatkaman**: data curation, investigation, methodology. **Sana Baghizadeh:** data curation, investigation, methodology. **Sarah Sadat Ehsani:** data curation, investigation, methodology. **Simin Lesan:** supervision, validation. **Hooman Ebrahimi:** supervision, validation.

## Funding

No funding was received for this manuscript.

## Conflicts of Interest

The authors declare no conflicts of interest.

## General Statement

*Transparency Statement*. The lead author, Reyhaneh Shoorgashti, affirms that this manuscript is an honest, accurate, and transparent account of the study being reported; that no important aspects of the study have been omitted; and that any discrepancies from the study as planned (and, if relevant, registered) have been explained.

## Supporting information


**Supporting Information** Additional supporting information can be found online in the Supporting Information section. Table S1 provides the full database‐specific search strategies, including MeSH terms and free‐text keywords used for MRSA, CAP, and bactericidal activity. Table S2 presents the modified Toxicological Data Reliability Assessment Tool (ToxRTool) scoring criteria used for risk of bias evaluation. Table S3 summarizes the findings of included in vitro studies investigating the effects of CAP on MRSA biofilms across different surface types. Table S4 provides a detailed overview of CAP instrumentation and operational parameters reported in the included studies. Table S5 presents the item‐level risk of bias assessments for all included studies based on the modified ToxRTool framework.

## Data Availability

The data that support the findings of this study are available in the Supporting Information of this article. Also, all authors have read and approved the final version of the manuscript. Dr. Reyhaneh Shoorgashti had full access to all of the data in this study and takes complete responsibility for the integrity of the data and the accuracy of the data analysis.
