# Supplementary material for: Efficacy of Cold Atmospheric Plasma Against Methicillin‐Resistant Staphylococcus aureus Biofilms: A Systematic Review of In Vitro Studies
Source: Biomed Res Int. 2026 Jul 27;2026:4047432. doi: 10.1155/bmri/4047432 (PMC13404011; doi:10.1155/bmri/4047432)
Supplement: Supplementary file 1 — Supporting Information Additional supporting information can be found online in the Supporting Information section. Table S1 provides the full database‐specific search strategies, including MeSH terms and free‐text keywords used for MRSA, CAP, and bactericidal activity. Table S2 presents the modified Toxicological Data Reliability Assessment Tool (ToxRTool) scoring criteria used for risk of bias evaluation. Table S3 summarizes the findings of included in vitro studies investigating the effects of CAP on MRSA biofilms across different surface types. Table S4 provides a detailed overview of CAP instrumentation and operational parameters reported in the included studies. Table S5 presents the item‐level risk of bias assessments for all included studies based on the modified ToxRTool framework. [file BMRI-2026-4047432-s001.docx]

**Supplementary Materials:**

Table 1. Database-specific search strategies developed using MeSH terms and free-text keywords for MRSA, cold atmospheric plasma (CAP), and bactericidal activity.

| Data Source | Search Query | Results |
| --- | --- | --- |
| PubMed | ((cold plasma[MeSH Terms]) OR (nonthermal plasma[MeSH Terms]) OR (non-equilibrium plasma) OR (Dielectric-barrier discharge plasma) OR (DBD plasma) OR (Atmospheric-pressure plasma) OR (normal pressure plasma) OR (Atmospheric Cold Plasma)) AND ((Methicillin Resistant Staphylococcus aureus) OR (Staphylococcus aureus) OR (MRSA)) | 261 |
| Embase | ('cold plasma'/exp OR 'cold plasma' OR (('cold'/exp OR cold) AND ('plasma'/exp OR plasma)) OR 'nonthermal plasma'/exp OR 'nonthermal plasma' OR (nonthermal AND ('plasma'/exp OR plasma)) OR 'non-equilibrium plasma' OR ('non equilibrium' AND ('plasma'/exp OR plasma)) OR 'dielectric-barrier discharge plasma' OR ('dielectric barrier' AND ('discharge'/exp OR discharge) AND ('plasma'/exp OR plasma))) AND ('methicillin resistant staphylococcus aureus'/exp OR 'methicillin resistant staphylococcus aureus' OR (('methicillin'/exp OR methicillin) AND resistant AND ('staphylococcus'/exp OR staphylococcus) AND aureus) OR 'staphylococcus aureus'/exp OR 'staphylococcus aureus' OR (('staphylococcus'/exp OR staphylococcus) AND aureus) OR 'mrsa'/exp OR mrsa) | 356 |
| Scopus | TITLE-ABS-KEY ( ( ( cold AND plasma ) OR ( nonthermal AND plasma ) OR ( non-equilibrium AND plasma ) OR ( dielectric-barrier AND discharge AND plasma ) OR ( dbd AND plasma ) OR ( atmospheric-pressure AND plasma ) OR ( atmospheric AND cold AND plasma ) ) AND ( ( methicillin AND resistant AND staphylococcus AND aureus ) OR ( staphylococcus AND aureus ) OR ( mrsa ) ) ) | 581 |
| ScienceDirect | ("cold plasma" OR "nonthermal plasma") AND ("Methicillin Resistant Staphylococcus aureus" OR "MRSA") | 171 |
| Google Scholar | allintitle: ("cold plasma" OR "nonthermal plasma" OR "non-equilibrium plasma" OR "Dielectric-barrier discharge plasma" OR "DBD plasma" OR "Atmospheric-pressure plasma") AND ("Methicillin Resistant Staphylococcus aureus" OR "MRSA") | 10 |

Table 2. Modified Toxicological Data Reliability Assessment Tool (ToxRTool) Scoring Criteria.

| Domain | Low Risk | Moderate Risk | High Risk |
| --- | --- | --- | --- |
| Test Substance Characterization | CAP device and treatment parameters are fully described | Some parameters are missing or incompletely reported | Major treatment characteristics absent |
| Test System Characterization | Strain, substrate, biofilm growth conditions, and maturity are adequately described | One or more characteristics are incompletely described | Critical characteristics absent |
| Study Design | Controls, replication, and procedures are clearly described | Minor reporting deficiencies | Major design limitations or inadequate reporting |
| Results Documentation | Outcomes clearly reported with sufficient detail | Some reporting deficiencies | Major reporting deficiencies |
| Data Plausibility | Results consistent with methods and biologically plausible | Minor inconsistencies | Major inconsistencies or implausible findings |

Table 3. The summary of findings from in vitro studies analyzing CAP on MRSA biofilms on different surfaces

| **Surface** | | **Author/ year/ ref** | **Country of Origin** | **Aim of study** | **Device** | **Surface** | **MRSA strains** | **Biofilm maturity confirmation** | **MRSA Microbial load (Log10) (Before)** | **MRSA Microbial load (After)** |
| --- | --- | --- | --- | --- | --- | --- | --- | --- | --- | --- |
| **Tissue models** | | Blaise 2024 ^1^ | France | To explore the CAP effects on infected wounds | CAP | Human bioengineered 3D models | S. aureus USA300 | Multiphoton microscopy  Confocal scanning laser microscopy | 10^8^ CFU/mL | **No treatment groups** mean bacterial count log₁₀ in CFU/mg: 7.50 ± 0.09  **Control groups** (helium) mean bacterial count log₁₀ in CFU/mg: 7.24 ± 0.27  **CAP groups** mean bacterial count log₁₀ in CFU/mg: 6.67 ± 0.42 |
|  |  | de Oliveira 2021 ^2^ | Brazil | To explore the CAP effects on wound-related multispecies and monospecies biofilms | CAP | Collagen membranes (Multispecies Biofilm) | S. aureus ATCC 33591 | SEM | 1.7 × 10⁸ CFU/mL (8.23 log CFU/mL) | **No-treatment:** 1.7×10⁸ CFU/mL (log 10 = 8.23 log CFU/mL)  **After 1 min CAP exposure:** 1.1 × 10^8^ CFU/mL (log 10 = 8.04 log CFU/mL)  **After 3 min CAP exposure:** 3.7 × 10^7^ CFU/mL (log 10 = 7.57 log CFU/mL)  **After 5 min CAP exposure:** 2.0 × 10^7^ CFU/mL (log 10 = 7.30 log CFU/mL)  **After 7 min CAP exposure:** 4.0 × 10^7^ CFU/mL (log 10 = 7.60 log CFU/mL) |
| **Bacterial culture** | | Lunder 2024 ^3^ | Slovenia | To explore the CAP potential for surface disinfection | CAP | Bacterial culture | S. aureus ATCC 43300 | Fluorescence microscopy | 1.5 × 10⁸ CFU/cm² (8.18 log CFU/cm²) | Log10 24h reduction rate:  **After 1 min CAP exposure:** 3.54 CFU/cm²  **After 2 min CAP exposure:** 1.46 CFU/cm²  **After 3 min CAP exposure:** 0.59 CFU/cm² |
|  |  |  |  |  |  |  |  |  | Log10 24h biofilm: 5.68 CFU/cm² | Log10 48h reduction rate:  **After 1 min CAP exposure:** 5.37 CFU/cm²  **After 2 min CAP exposure:** 2.29 CFU/cm²  **After 3 min CAP exposure:** 1.59 CFU/cm² |
|  |  |  |  |  |  |  |  |  | Log10 48h biofilm: 6.61 CFU/cm² | Log10 72h reduction rate:  **After 1 min CAP exposure:** 4.91 CFU/cm²  **After 2 min CAP exposure:** 2.06 CFU/cm²  **After 3 min CAP exposure:** 1.55 CFU/cm² |
|  |  |  |  |  |  |  |  |  | Log10 72h biofilm: 6.80 CFU/cm² |  |
|  |  | Thana 2019 ^4^ | Thailand | To explore the effects of an air plasma jet and the different factors that affect ROS | CAP jet | Bacterial culture | S. aureus ATCC33591 | *NA* | > 10^6^ CFUs  (> 6 log CFUs) | **Complete bacterial clearance** (After 120s exposure) |
|  |  | Mohd Nasir 2016 ^5^ | Malaysia | To explore the effects of two CAP devices on MRSA and P. aeruginosa | Parallel-plate DBD  CGCD | Bacterial culture | *NA* | *NA* | Parallel-plate DBD: 9.5 log10  CGCD: 9 log10 | **Parallel-plate DBD:** 1 log10 reduction (After 30 min plasma exposure)  **CGCD:** 9 log10 reduction (After 30 min plasma exposure) |
| **Well plates** | | de Oliveira 2021 ^2^ | Brazil | To explore the CAP effects on wound-related multispecies and monospecies biofilms | CAP | 96-well plates (Monospecies Biofilm) | S. aureus ATCC33591 | SEM | 1.7 × 10⁸ CFU/mL (8.23 log CFU/mL) | **No-treatment:**  6.4 × 10^8^ CFU/mL (log 10 = 8.81 log CFU/mL)  **After 1 min CAP exposure:** 8.0 × 10⁶ CFU/mL (log 10 = 6.90 log CFU/mL)  **After 3 min CAP exposure:** 3.0 × 10⁶ CFU/mL (log 10 = 6.48 log CFU/mL)  **After 5 min CAP exposure:** 8.0 × 10⁵ CFU/mL (log 10 = 5.90 log CFU/mL)  **After 7 min CAP exposure:** 1.4 × 10⁶ CFU/mL (log 10 = 6.15 log CFU/mL) |
|  |  | Fallon 2021 ^6^ | Ireland | To explore the CAP effects on hospital surface disinfection | CAPP | 96-well plates | S. aureus BH1CC | Confocal scanning laser microscopy | 10^7^ CFU/mL (7 log CFU/mL) | **Biofilm viability on Polystyrene Surfaces after CAPP exposure:**  96-well plates: 30% |
|  |  | Guo 2021 ^7^ | USA | To explore the CAP-induced oxidative damage to proteins in MRSA biofilms | CAPP | 96-well plates with single-sided adhesive silica films | S. aureus ATCC33591 | *NA* | ≈ 2 × 10⁷ CFU/mL (≈ 7.3 log CFU/mL) | Reduction rate:  0.5 log10 steps (After 2 min CAPP exposure)  Less than 2 log10 steps (After 4 min CAPP exposure)  3.5 log10 steps (After 6 min CAPP exposure) |
|  |  | Yang 2021^8^ | China | To explore the effects of PAS and antibiotic combination in in vitro and infected murine models | PAS | 96-well plates with single-sided adhesive silica films | S. aureus ATCC33591 | *NA* | *NA* | **No PAS- No antibiotic (Control group): ~** 8 log10  **PAS treatment alone: ~** 7.1 log10  **0.625 mg/mL vancomycin:** **~** 6.9 log10  **PAS + 0.625 mg/mL vancomycin:** < 2 log10  **1.25 mg/mL vancomycin: ~** 6.8 log10  **PAS + 1.25 mg/mL vancomycin:** < 2 log10  **0.3125 mg/mL Rifampicin treatment: ~** 4.5 log10  **PAS + 0.3125 mg/mL Rifampicin treatment:** < 2 log10  **0.625 mg/mL Rifampicin treatment: ~** 4.6 log10  **PAS + 0.625 mg/mL Rifampicin treatment:** < 2 log10 |
|  |  | Guo 2019 ^9^ | USA | To explore the effects of surface discharge plasma inactivating biofilms of MRSA, and the correlation between plasma-generated reactive species and the inactivation efficiency of biofilms | Surface discharge plasma | 24-well plates with single-sided adhesive silica films | S. aureus ATCC 33591 | *NA* | 7.5 log10 CFU | **Inactivated MRSA in log10:**  **3min-He+1% air plasma:** 0.8 log10  **3min-Ar+1% air plasma:** 2 log10  **3min-Ar+1% synthetic air plasma:** 2 log10  **3min-Ar+1% natural air plasma:** 2 log10  **5min-He+1% air plasma:** 2.3 log10  **5min-Ar+1% air plasma:** 5 log10  **5min-Ar+1% synthetic air plasma:** 3.7 log10  **5min-Ar+1% natural air plasma:** 4.8 log10 |
|  |  | Brun 2018 ^10^ | Italy | To explore the CAPP effects on MRSA and P. aeruginosa | Radiofrequency plasma jet | 96-well plates | S. aureus ATCC 33592 | *NA* | 10^5^ CFU/mL (5 log CFU/mL) | ~1.5–2.0 log₁₀ CFU/mL reduction (After 2min plasma exposure) |
|  |  | Miletić 2014 ^11^ | Serbia | To explore the effects of the plasma needle on MRSA biofilm | Plasma needle device | 96-well plates | *NA* | *NA* | Three different inoculum size:  10^4^ to 10^6^ CFU per well  (4-6 log) | **Almost complete inhibition** (After 120s exposure) |
|  |  | Joshi 2010 ^12^ | USA | To explore the effects of floating-electron-DBD on bacterial forms and surface disinfections | Floating-electrode DBD | 96-well plates | S. aureus USA300  S. aureus USA400 | *NA* | ~10⁶–10⁷ CFU per well | **Almost complete inactivation** (After 120s indirect plasma exposure) |
| **Hospital surfaces** | **Glass** | Fallon 2021 ^6^ | Ireland | To explore the CAP effects on hospital surface disinfection | CAPP | Glass | S. aureus BH1CC | Confocal scanning laser microscopy | 10^7^ CFU/mL (7 log CFU/mL) | **Biofilm viable bacteria after 24h of biofilm growth:**  43% (untreated group)  14% (After 1.5 min CAPP exposure)  **Biofilm viable bacteria after 48h of biofilm growth:**  43% (untreated group)  7% (After 1.5 min CAPP exposure)  **Biofilm viable bacteria after 72h of biofilm growth:**  56% (untreated group)  18% (After 1.5 min CAPP exposure) |
|  |  | Cotter 2011 ^13^ | Ireland | To explore the DBD effects on the S. epidermidis and MRSA biofilms’ surface disinfection | DBD | Glass coverslips | S. aureus BH1CC | *NA* | 9.1 log10 CFU/cm^2^ | 4 log10 reduction (After 60min plasma exposure)  5.5 log10 reduction (After 90min plasma exposure) |
|  |  | Joshi 2010 ^12^ | USA | To explore the effects of floating-electron-DBD on bacterial forms and surface disinfections | Floating-electrode DBD | Glass coverslips | S. aureus USA300  S. aureus USA400 | *NA* | ~10⁶–10⁷ CFU per well | **Complete bacterial clearance** (After 120s direct plasma exposure) |
|  | **Polyethylene** | Kelar Tučeková 2021 ^14^ | Czech Republic | To explore the effects of plasma-activated water vapor/aerosol generated via MSDBD on bacterial biofilm decontamination | MSDBD | Polypropylene nonwoven textile | S. aureus ATCC 43300 | *NA* | 1.5 × 10⁸ CFU/cm² (8.18 log CFU/cm²) | **Minimum reduction rate:** 2.9 log10 steps (After 150 s exposure)  **Maximum reduction rate:** 4.8 log10 steps (After 150 s exposure) |
|  |  | Daeschlein 2012 ^15^ | Germany | To explore the CAP susceptibility of various clinical bacterial strains | DBD | Polyethylene | HA-MRSA (hospital-associated)  S. aureus USA300  LA-MRSA (livestock-associated, STD 1398) | Confocal laser scan microscopy | *NA* | 3 to 5 log steps bacterial reduction |
|  | **Stainless steel** | Fallon 2021 ^6^ | Ireland | To explore the CAP effects on hospital surface disinfection | CAPP | Stainless steel coupon  Powder-coated steel coupon | S. aureus BH1CC | Confocal scanning laser microscopy | 10^7^ CFU/mL (7 log CFU/mL) | **Biofilm viability on Steel Surfaces after CAPP exposure:**  Stainless steel coupon: 56%  Powder-coated steel coupon: 70% |
|  |  | Lis 2018 ^16^ | Germany | To explore the CAPP effects on reducing pathogens and the influence of organic material | Surface microdischarge | Stainless steel metal plates | OXA resistance phenotype  mecA resistant genes | Fluorescence microscopy | 10^6^ CFU/mL (6 log CFU/mL) | 1.80 ± 0.59 log10 reduction (After 5min plasma exposure)  2.51 ± 0.31 log10 reduction (After 10min plasma exposure)  3.38 ± 0.62 log10 reduction (After 20min plasma exposure) |
|  |  | Cahill 2017 ^17^ | Ireland | To explore the CAPP effects on decontaminating two hospital surfaces | Multi-jet CAPP | Stainless steel | S. aureus ATCC 43300 | *NA* | 9.2 log10 CFU/mL | 6.21 log10 reduction (After 45 s plasma exposure) |
|  |  | Daeschlein 2012 ^15^ | Germany | To explore the CAP susceptibility of various clinical bacterial strains | DBD | Stainless steel | HA-MRSA (hospital-associated)  S. aureus USA300  LA-MRSA (livestock-associated, STD 1398) | Confocal laser scan microscopy | *NA* | **Complete bacterial clearance** (After 1 min exposure) |
|  | **Linoleum** | Fallon 2021 ^6^ | Ireland | To explore the CAP effects on hospital surface disinfection | CAPP | Linoleum flooring Surface | S. aureus BH1CC | Confocal scanning laser microscopy | 10^7^ CFU/mL (7 log CFU/mL) | **Biofilm viability on Linoleum Surface after CAPP exposure:**  Flooring coupon: 30% |
|  | **Fabric** | Cahill 2017 ^17^ | Ireland | To explore the CAPP effects on decontaminating two hospital surfaces | Multi-jet CAPP | Mattress | S. aureus ATCC 43300 | *NA* | 9.2 log10 CFU/mL | 4.14 log10 reduction (After 45 s plasma exposure) |

**Abbreviation: CAPP,** Cold Atmospheric Pressure Plasma**; CAP,** Cold Atmospheric Plasma**; CFU,** Colony Forming Unit**; CFU/cm²,** Colony Forming Units per Square Centimeter**; CFU/mg,** Colony Forming Units per Milligram**; CFU/mL,** Colony Forming Units per Milliliter**; CGCD,** Cold Gas Capacitively Coupled Discharge**; DBD,** Dielectric Barrier Discharge**; Log₁₀,** Logarithm to base 10**; MSDBD,** Multi-Hollow Surface Dielectric Barrier Discharge**; *NA*,** Not Available**; PAS,** Plasma-Activated Solution**; P. aeruginosa,** *Pseudomonas aeruginosa***; MRSA,** Methicillin-Resistant *Staphylococcus aureus***; S. epidermidis,** *Staphylococcus epidermidis***; S. aureus,** *Staphylococcus aureus***; SEM,** Scanning Electron Microscopy.

Table 4. The summary of findings from the CAP Instrumentation and Settings of in vitro studies analyzing CAP on MRSA biofilms on different surfaces

| **Author/ year/ ref** | **CAP System** | **CAP Utilized Gas** | **Applied Voltage** | **Frequency of Discharge** | **Input CAP power in Watt** | **Distance from Sample (mm)** | **Exposure Duration** |
| --- | --- | --- | --- | --- | --- | --- | --- |
| Blaise O 2024 ^1^ | Dielectric Helium CAP jet | He | 32 kV | *NA* | 90 ± 10 mW | 4–6 mm | 2 min |
| Lunder M 2024 ^3^ | Gliding arc plasma jet | Atmospheric air | 20 V | *NA* | 35 W | 1-10 mm | 1, 2, 3 min |
| de Oliveira 2021 ^2^ | DBD-based plasma jet | He | 12 kV | *NA* | *NA* | 15 mm | 1, 3, 5, and 7 min |
| Fallon 2021 ^6^ | Plasma jet | Air | 30 kV | 21-23 kHz | *NA* | 15 mm | 0.5 and 1.5 min |
| Guo 2021 ^7^ | Surface discharge plasma | He+1% synthetic air | 7.44 kV | 20 kHz | 0.2 W/cm2 | 8 *±* 0.2 mm | 2 min to 4 min |
| Kelar Tučeková 2021 ^14^ | MSDBD | Ambient air  Water vapor | *NA* | 25–27 kHz | Ambient air: 2.9 W/cm^2^  Water vapor: 6.2 W/cm^2^ | 20, 30, and 40 cm | 0.5-2.5 min |
| Yang 2021^8^ | PAS | *NA* | *NA* | *NA* | *NA* | *NA* | 30 min |
| Guo 2019 ^9^ | Surface discharge plasma | He+1% synthetic air  Ar+1% synthetic air  Synthetic air  Natural air | 6.2-7.7 kV | *NA* | ~0.1 W/cm2 | 9 mm | 2 min |
| Thana 2019 ^4^ | Coaxial pulsed plasma jet | Ambient air | 2.52 kV | 2 kHz | *NA* | 1 mm | 1.5 min |
| Brun 2018 ^10^ | Radiofrequency plasma jet | He | 1 kV | 4.5 MHz | 0.4 W | 1.5 mm | 2 min |
| Lis 2018 ^16^ | Surface microdischarge | Ambient air | 10 kV | 2 kHz | *NA* | *NA* | 5, 10, and 20 min |
| Cahill 2017 ^17^ | Multi-jet CAPP | Dry air | 5 kV | 8 kHz | 15 W | 10 mm | 15, 20, 30, and 45 seconds |
| Mohd Nasir 2016 ^5^ | Parallel plate DBD  CGCD | Ambient air | DBD: 40 kV (AC)  CGCD: 9.9-11.6 kV (DC) | DBD: 50 Hz  CGCD: 8-15 kHz | DBD: ~25 mJ per cycle  CGCD: 0.05-0.25 W | DBD: 0.1-15 mm  CGCD: 6-10 mm | DBD: 1-60 min  CGCD: 1-30 min |
| Miletić 2014 ^11^ | Plasma needle device | He | *NA* | 13.56 MHz | 0.15, 0.9, 1.6 W | 3 mm | 30, 60 and 120 s |
| Daeschlein 2012 ^15^ | DBD | Air | 14 kV | 100 and 400 Hz | 167–237 mW | 2 mm | 1, 2, and 3 min |
| Cotter 2011 ^13^ | DBD | Air | 10 kV | 25 kHz | *NA* | 21 mm | 0-90 min |
| Joshi 2010 ^12^ | Floating-electrode DBD | Air | 120 V | *NA* | 0.13 W/cm^2^ | ~3 mm | 0-2.5 min |

**Abbreviation: CAPP,** Cold Atmospheric Pressure Plasma**; CAP,** Cold Atmospheric Plasma**; CFU,** Colony Forming Unit**; CFU/cm²,** Colony Forming Units per Square Centimeter**; CFU/mg,** Colony Forming Units per Milligram**; CFU/mL,** Colony Forming Units per Milliliter**; CGCD,** Cold Gas Capacitively Coupled Discharge**; DBD,** Dielectric Barrier Discharge**; Log₁₀,** Logarithm to base 10**; MSDBD,** Multi-Hollow Surface Dielectric Barrier Discharge**; *NA*,** Not Available**; PAS,** Plasma-Activated Solution**; P. aeruginosa,** *Pseudomonas aeruginosa***; MRSA,** Methicillin-Resistant *Staphylococcus aureus***; S. epidermidis,** *Staphylococcus epidermidis*.

Table 5. Item-level risk-of-bias assessments of included studies using the modified ToxRTool framework.

| **Author/ year/ ref** | **Test Substance** | **Test System** | **Study Design** | **Results Documentation** | **Data Plausibility** | **Overall Risk** |
| --- | --- | --- | --- | --- | --- | --- |
| Blaise O 2024 ^1^ | Moderate | Low | Low | Low | Low | Moderate |
| Lunder M 2024 ^3^ | Moderate | Low | Low | Low | Low | Moderate |
| de Oliveira 2021 ^2^ | Moderate | Low | Low | Low | Low | Moderate |
| Fallon 2021 ^6^ | Moderate | Low | Low | Low | Low | Moderate |
| Guo 2021 ^7^ | Low | Moderate | Low | Low | Low | Moderate |
| Kelar Tučeková 2021 ^14^ | Moderate | Moderate | Low | Low | Low | Moderate |
| Yang 2021^8^ | High | High | Low | Low | Low | High |
| Guo 2019 ^9^ | Moderate | Moderate | Low | Low | Low | Moderate |
| Thana 2019 ^4^ | Moderate | Moderate | Low | Low | Low | Moderate |
| Brun 2018 ^10^ | Low | Moderate | Low | Low | Low | Moderate |
| Lis 2018 ^16^ | Moderate | Low | Low | Low | Low | Moderate |
| Cahill 2017 ^17^ | Low | Moderate | Low | Low | Low | Moderate |
| Mohd Nasir 2016 ^5^ | Low | Moderate | Low | Low | Low | Moderate |
| Miletić 2014 ^11^ | Moderate | Moderate | Low | Low | Low | Moderate |
| Daeschlein 2012 ^15^ | Low | Low | Low | Low | Low | Low |
| Cotter 2011 ^13^ | Moderate | Moderate | Low | Low | Low | Moderate |
| Joshi 2010 ^12^ | Moderate | Moderate | Low | Low | Low | Moderate |

1. Blaise O, Duchesne C, Capuzzo E, Nahori MA, Fernandes J, Connor MG, et al. Infected wound repair correlates with collagen I induction and NOX2 activation by cold atmospheric plasma. *NPJ Regen Med*. 2024;**9**(1):28. [PubMed ID: PMC11447178]. <https://doi.org/10.1038/s41536-024-00372-0>.

2. Oliveira M, Lima G, Nishime T, Gontijo A, Menezes B, Caliari M, et al. Inhibitory Effect of Cold Atmospheric Plasma on Chronic Wound-Related Multispecies Biofilms. *Applied Sciences*. 2021;**11**:5441. <http://doi.org/10.3390/app11125441>.

3. Lunder M, Dahle S, Fink R. Cold atmospheric plasma for surface disinfection: a promising weapon against deleterious meticillin-resistant Staphylococcus aureus biofilms. *J Hosp Infect*. 2024;**143**:64-75. <https://doi.org/10.1016/j.jhin.2023.10.014>.

4. Thana P, Wijaikhum A, Poramapijitwat P, Kuensaen C, Meerak J, Ngamjarurojana A, et al. A compact pulse-modulation cold air plasma jet for the inactivation of chronic wound bacteria: development and characterization. *Heliyon*. 2019;**5**(9):e02455. [PubMed ID: PMC6819795]. <https://doi.org/10.1016/j.heliyon.2019.e02455>.

5. Mohd Nasir N, Lee BK, Yap SS, Thong KL, Yap SL. Cold plasma inactivation of chronic wound bacteria. *Archives of Biochemistry and Biophysics*. 2016;**605**:76-85. <https://www.sciencedirect.com/science/article/pii/S0003986116300935>.

6. Fallon M, Kennedy S, Kumar S, Daniels S, Humphreys H. The potential use of a cold atmospheric plasma jet for decontamination of hospital surfaces. A pilot study. *Plasma Medicine*. 2021;**11**(1):15-30. <https://www.embase.com/search/results?subaction=viewrecord&id=L2007613452&from=export>

<http://dx.doi.org/10.1615/PlasmaMed.2021037267>.

7. Guo L, Yang L, Qi Y, Niyazi G, Huang L, Gou L, et al. Cold Atmospheric-Pressure Plasma Caused Protein Damage in Methicillin-Resistant Staphylococcus aureus Cells in Biofilms. *Microorganisms*. 2021;**9**(5). [PubMed ID: PMC8156483]. <https://doi.org/10.3390/microorganisms9051072>.

8. Yang L, Niyazi G, Qi Y, Yao Z, Huang L, Wang Z, et al. Plasma-activated saline promotes antibiotic treatment of systemic methicillin-resistant staphylococcus aureus infection. *Antibiotics*. 2021;**10**(8):1018. <https://www.embase.com/search/results?subaction=viewrecord&id=L2013511229&from=export>

<http://dx.doi.org/10.3390/antibiotics10081018>

<https://mdpi-res.com/d_attachment/antibiotics/antibiotics-10-01018/article_deploy/antibiotics-10-01018.pdf?version=1629622158>.

9. Guo L, Xu R, Liu D, Qi Y, Guo Y, Wang W, et al. Eradication of methicillin-resistant Staphylococcus aureus biofilms by surface discharge plasmas with various working gases. *Journal of Physics D: Applied Physics*. 2019;**52**(42):425202. <https://www.scopus.com/inward/record.uri?eid=2-s2.0-85071453451&doi=10.1088%2F1361-6463%2Fab32c9&partnerID=40&md5=50b4ca37491d8a86373512bcb8e3e3d5>.

10. Brun P, Bernabè G, Marchiori C, Scarpa M, Zuin M, Cavazzana R, et al. Antibacterial efficacy and mechanisms of action of low power atmospheric pressure cold plasma: membrane permeability, biofilm penetration and antimicrobial sensitization. *Journal of Applied Microbiology*. 2018;**125**(2):398-408. <https://www.embase.com/search/results?subaction=viewrecord&id=L623019262&from=export>

<http://dx.doi.org/10.1111/jam.13780>.

11. Miletić M, Vuković D, Živanović I, Dakić I, Soldatović I, Maletić D, et al. Inhibition of methicillin resistant staphylococcus aureus by a plasma needle. *Central European Journal of Physics*. 2014;**12**(3):160-7. <https://www.scopus.com/inward/record.uri?eid=2-s2.0-84937523731&doi=10.2478%2Fs11534-014-0437-z&partnerID=40&md5=0db022378912c467bb28beccb17f4a73>.

12. Joshi SG, Paff M, Friedman G, Fridman G, Fridman A, Brooks AD. Control of methicillin-resistant Staphylococcus aureus in planktonic form and biofilms: a biocidal efficacy study of nonthermal dielectric-barrier discharge plasma. *American journal of infection control*. 2010;**38**(4):293-301.

13. Cotter JJ, Maguire P, Soberon F, Daniels S, O'Gara JP, Casey E. Disinfection of meticillin-resistant Staphylococcus aureus and Staphylococcus epidermidis biofilms using a remote non-thermal gas plasma. *Journal of Hospital Infection*. 2011;**78**(3):204-7. <https://www.embase.com/search/results?subaction=viewrecord&id=L51435556&from=export>

<http://dx.doi.org/10.1016/j.jhin.2011.03.019>.

14. Kelar Tučeková Z, Vacek L, Krumpolec R, Kelar J, Zemánek M, Černák M, et al. Multi-Hollow Surface Dielectric Barrier Discharge for Bacterial Biofilm Decontamination. *Molecules (Basel, Switzerland)*. 2021;**26**(4):910. <https://www.embase.com/search/results?subaction=viewrecord&id=L634266153&from=export>

<http://dx.doi.org/10.3390/molecules26040910>.

15. Daeschlein G, Scholz S, Emmert S, von Podewils S, Haase H, von Woedtke T, et al. Plasma medicine in dermatology: Basic antimicrobial efficacy testing as prerequisite to clinical plasma therapy. *Plasma Medicine*. 2012;**2**(1-3):33-69. <https://www.embase.com/search/results?subaction=viewrecord&id=L600202636&from=export>

<http://dx.doi.org/10.1615/PlasmaMed.2014006217>.

16. Lis KA, Kehrenberg C, Boulaaba A, von Köckritz-Blickwede M, Binder S, Li Y, et al. Inactivation of multidrug-resistant pathogens and Yersinia enterocolitica with cold atmospheric-pressure plasma on stainless-steel surfaces. *International Journal of Antimicrobial Agents*. 2018;**52**(6):811-8. <https://www.embase.com/search/results?subaction=viewrecord&id=L2001278357&from=export>

<http://dx.doi.org/10.1016/j.ijantimicag.2018.08.023>.

17. Cahill OJ, Claro T, Cafolla AA, Stevens NT, Daniels S, Humphreys H. Decontamination of hospital surfaces with multijet cold plasma: A method to enhance infection prevention and control? *Infection Control and Hospital Epidemiology*. 2017;**38**(10):1182-7. <https://www.embase.com/search/results?subaction=viewrecord&id=L618474930&from=export>

<http://dx.doi.org/10.1017/ice.2017.168>.
